# Increased level of serum leucine-rich-alpha-2-glycoprotein 1 in patients with clear cell renal cell carcinoma

**DOI:** 10.1186/s12894-024-01481-0

**Published:** 2024-04-24

**Authors:** Shotaro Nakanishi, Masato Goya, Tetsuji Suda, Tomoko Yonamine, Ai Sugawa, Seiichi Saito

**Affiliations:** 1 Department of Urology, Graduate School of Medicine, University of the Ryukyus, 207 Uehara, Nishihara, 903-0215 Okinawa Japan; 2 Chubu Tokusyukai Hospital, Kitanakagusuku, 801 higa, 901-2392 Okinawa Japan

**Keywords:** LRG1, Leucine-rich-alpha-2-glycoprotein 1, Clear cell renal cell carcinoma, Serum marker

## Abstract

**Background:**

Currently, no useful serum markers exist for clear cell renal cell carcinoma (ccRCC), making early detection challenging as diagnosis relies solely on imaging tests. Radiation exposure is also a concern due to multiple required CT examinations during treatment. Renal cell carcinoma (RCC) histological types include ccRCC and non-clear cell RCC (non-ccRCC); however, treatment response to medications varies which necessitates accurate differentiation between the two. Therefore, we aimed to identify a novel serum marker of RCC. Increased LRG1 expression in the serum has been demonstrated in multiple cancer types. However, the expression of LRG1 expression in the serum and cancer tissues of patients with RCC has not been reported. Since ccRCC is a hypervascular tumor and LRG1 is capable of accelerating angiogenesis, we hypothesized that the LRG1 levels may be related to ccRCC. Therefore, we examined LRG1 expression in sera from patients with RCC.

**Methods:**

Using an enzyme-linked immunosorbent assay, serum levels of leucine-rich-alpha-2-glycoprotein 1 (LRG1) were measured in 64 patients with ccRCC and 22 patients non-ccRCC who underwent radical or partial nephrectomy, as well as in 63 patients without cancer.

**Results:**

Median values of serum LRG1 and their inter-quartile ranges were 63.2 (42.8–94.2) µg/mL in ccRCC, 23.4 (17.7–29.6) µg/mL in non-ccRCC, and 36.0 (23.7–56.7) µg/mL in patients without cancer, respectively (ccRCC vs. non-ccRCC or patients without cancer: *P* < 0.001). C-reactive protein (CRP) levels (*P* = 0.002), anemia (*P* = 0.037), hypercalcemia (*P* = 0.023), and grade (*P* = 0.031) were independent predictors of serum LRG1 levels in ccRCC. To assess diagnostic performance, the area under the receiver operating characteristic curve of serum LRG1 was utilized to differentiate ccRCC from non-cancer and non-ccRCC, with values of 0.73 (95% CI, 0.64–0.82) and 0.91 (95% CI, 0.82–0.96), respectively.

**Conclusions:**

LRG1 served as a serum marker associated with inflammation, indicated by CRP, anemia, hypercalcemia, and malignant potential in ccRCC. Clinically, serum LRG1 levels may assist in differentiating ccRCC from non-ccRCC with excellent diagnostic accuracy.

**Supplementary Information:**

The online version contains supplementary material available at 10.1186/s12894-024-01481-0.

## Background

Renal cell carcinoma (RCC) is the most common type of kidney cancer, accounting for approximately 2 − 3% of all malignancies [1]. Urological cancer ranks as the second leading cause of death among all urological cancers. Despite increased RCC diagnoses over the years, many patients (25-30%) still present with distant metastases at diagnosis, with metastasis developing in about 30% of the remaining patients [2]. Surgery proves most effective for localized RCC, while molecular-targeted therapies, including tyrosine kinase inhibitors (TKI) and immuno-oncology (IO) drugs, have improved overall survival, particularly for locally advanced and metastatic RCC (mRCC) [3, 4]. Although TKIs and nivolumab have led to innovations in treating advanced clear cell renal cell carcinoma (ccRCC), prognosis remains generally poor [5]. Selecting treatment for mRCC would ideally involve determining whether the renal tumor is ccRCC or non-ccRCC without biopsy or excision, as most patients with ccRCC do not benefit from IO drugs, although about 30% of patients with mRCC achieve long-term survival [4]. Therefore, detecting RCC at a localized or less advanced stage may contribute to a more favorable prognosis.

Leucine-rich-2-glycoprotein (LRG) was first identified in the human serum [6], with leucine-rich-alpha-2-glycoprotein 1 (LRG1) identified subsequently as a member of the membrane-associated leucine-rich repeat (LRR) family. LRG1 plays roles in cell adhesion [7], migration [8], survival, and apoptosis [9, 10]. Recently, Wang et al. revealed that LRG1 was capable of accelerating angiogenesis via direct binding to the TGF-β accessory receptor endoglin to activate the Smad1/5/8 signalling pathway [11]. 

Increased LRG1 expression in the serum has been demonstrated in ovarian cancer [12], non-small cell lung cancer [13], gastric cancer [14], pancreatic cancer [15], and leukemia [16]. However, LRG1 expression in the serum and cancer tissues of patients with RCC has not been reported. Since ccRCC is a hypervascular tumor and LRG1 is capable of accelerating angiogenesis [11], we hypothesized that LRG1 levels may be related to ccRCC. Therefore, we examined LRG1 expression in sera from patients with RCC.

## Materials and methods

### Patients and samples

Eighty-six consecutive patients with RCC who underwent radical or partial nephrectomy between 2012 and 2017 at the Department of Urology, University of the Ryukyus Hospital, were studied. Serum samples were obtained from 64 patients with ccRCC, 22 with non-ccRCC, and 63 without cancer. The inclusion criteria for the 63 non-cancer patients were those with benign diseases who visited our department between 2012 and 2017. Their details are shown as Supplementary Table 1. All samples were collected within 3 months before surgery. Serum was aliquoted and frozen at -80 °C until use. Pathological stages of the specimens were assessed according to the TNM Classification of Malignant Tumors, 7th edition [17]. 

This study was approved by the internal review board of the University of the Ryukyus (No. 524), and informed consent was obtained from each patient. The study was conducted in accordance with the principles Declaration of Helsinki.

### Western blot analysis of LRG1 proteins

To eliminate serum immunoglobulin G, patient serum samples were pre-treated with protein G spin columns (Cosmo Bio Co., Ltd. Tokyo, Japan). Cultured RCC cells were lysed in NP40 cell lysis buffer (Life Technologies) supplemented with a protease inhibitor cocktail (Roche Diagnostics GmbH, Mannheim, Germany). Ten micrograms of serum proteins or 15 µg of cell lysis proteins were boiled in sample buffer and fractionated using Mini-PROTEAN TGX 10% gel electrophoresis (Bio-Rad Laboratories, Inc., Hercules, CA, USA). After protein transfer to PVDF membranes (Bio-Rad Laboratories, Inc.), the membranes were blocked with 5% BSA in TBS (Bio-Rad Laboratories, Inc.) containing 0.05% Tween-20. LRG1 antibody (Proteintech Group, Inc., Rosemont, IL) binding to the blot proteins was detected using HRP-linked anti-rabbit IgG (GE Healthcare UK, Ltd., Little Chalfront, UK), followed by chemiluminescence (Bio-Rad Laboratories, Inc.).

### Enzyme-linked immunosorbent assay

Serum levels of LRG1 in patients with RCC and patients without cancer were measured by enzyme-linked immunosorbent assay (ELISA) using a Human LRG Assay Kit (Immuno-Biological Laboratories Co., Ltd., Gunma, Japan) according to the manufacturer’s instructions. Absorbance was measured at 450 nm using a Model 680 microplate reader (Bio-Rad Laboratories, Inc.). All experiments were performed in duplicates.

### Statistical analysis

Statistical analyses were conducted using the JMP 12® (SAS Institute Inc., Cary, NC, USA). Statistical significance was determined using the nonparametric Mann-Whitney U test (which assessed differences among the three groups). The Spearman rank correlation coefficient was utilized to determine the correlation between clinical parameters and multiple regression analysis of multivariable analysis. A receiver operating characteristic (ROC) curve was generated to assess the diagnostic efficiency. A *P-*value < 0.05 was considered statistically significant.

### Data availability

Data supporting the study findings are available from the corresponding author upon reasonable request.

## Results

### Clinicopathological characteristics of the patients

The clinicopathological characteristics of 86 patients with ccRCC and non-ccRCC are summarized in Table 1. All patients with RCC underwent radical or partial nephrectomy. Among the 22 patients with non-ccRCC, 13 (59%) had papillary RCC and nine (41%) had chromophobe RCC. No significant differences were observed in the variables between the ccRCC and non-ccRCC patients.

**Table 1:** Clinicopathological characteristics of patients with RCC

	ccRCC (*N* = 64)	non ccRCC (*N* = 22)	p value
M / F	37 (58%) / 27 (42%)	14 (64%) / 8 (36%)	0.63
Age, median	62.5 (35-87)	60.5 (23-82)	0.49
TNM classification			
clinical T			0.42
1	50 (78%)	19 (86%)	
2	5 (8%)	2 (9%)	
3	9 14%)	1 (5%)	
4	0	0	
clinical N			0.06
0	64 (100%)	20 (90%)	
1	0	1 (5%)	
2	0	1 (5%)	
clinical M			0.97
0	58 (91%)	20 (90%)	
1	6 (9%)	2 (10%)	
Grading			0.18
1	8 (13%)	1 (5%)	
2	49 (76%)	14 (64%)	
3	6 (9%)	5 (22%)	
N.A.	1 (2%)	2 (9%)	
pathological T			0.13
1	47 (74%)	19 (86%)	
2	6 (9%)	0	
3	11 (17%)	3 (14%)	
4	0	0	
INF			0.15
a	47 (73%)	20 (90%)	
b	15 (24%)	2 (10%)	
c	0	0	
N.A.	2 (3%)	0	
v			0.31
0	46 (72%)	19 (86%)	
1	17 (16%)	3 (14%)	
N.A.	1 (2%)	0	
Tumor size (cm)	3.5 (2.23 - 5.85)	2.9 (2.23 - 4.55)	0.41
CRP (mg/dl)	0.13 (0.1-0.43)	0.1 (0.1-0.81)	0.90
LRG1 (ug/ml)	63.2 (42.8 - 94.2)	23.4 (17.7 - 29.6)	<0.0001

### Western blotting of serum LRG1

**Fig. 1:**
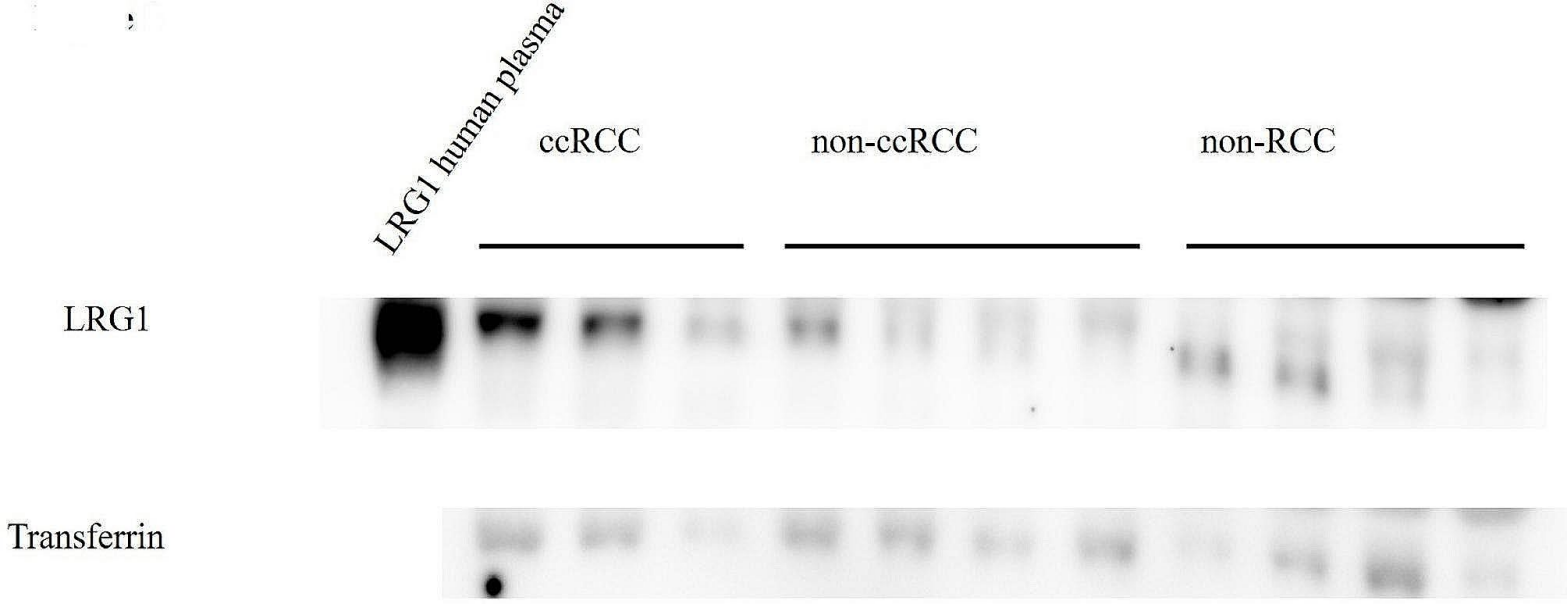
Western blotting was utilized to determine if LRG1 was present in RCC serum. Transferrin was utilized as an internal control. LRG1 expression was markedly higher in ccRCC as compared to non-ccRCC

Western blotting results demonstrated that the expression level of serum LRG1 in the patients with ccRCC was higher as compared to those with non-ccRCC or patients without cancer (Fig. 1).

### Measurement of LRG1 in patient serum by ELISA

**Fig. 2:**
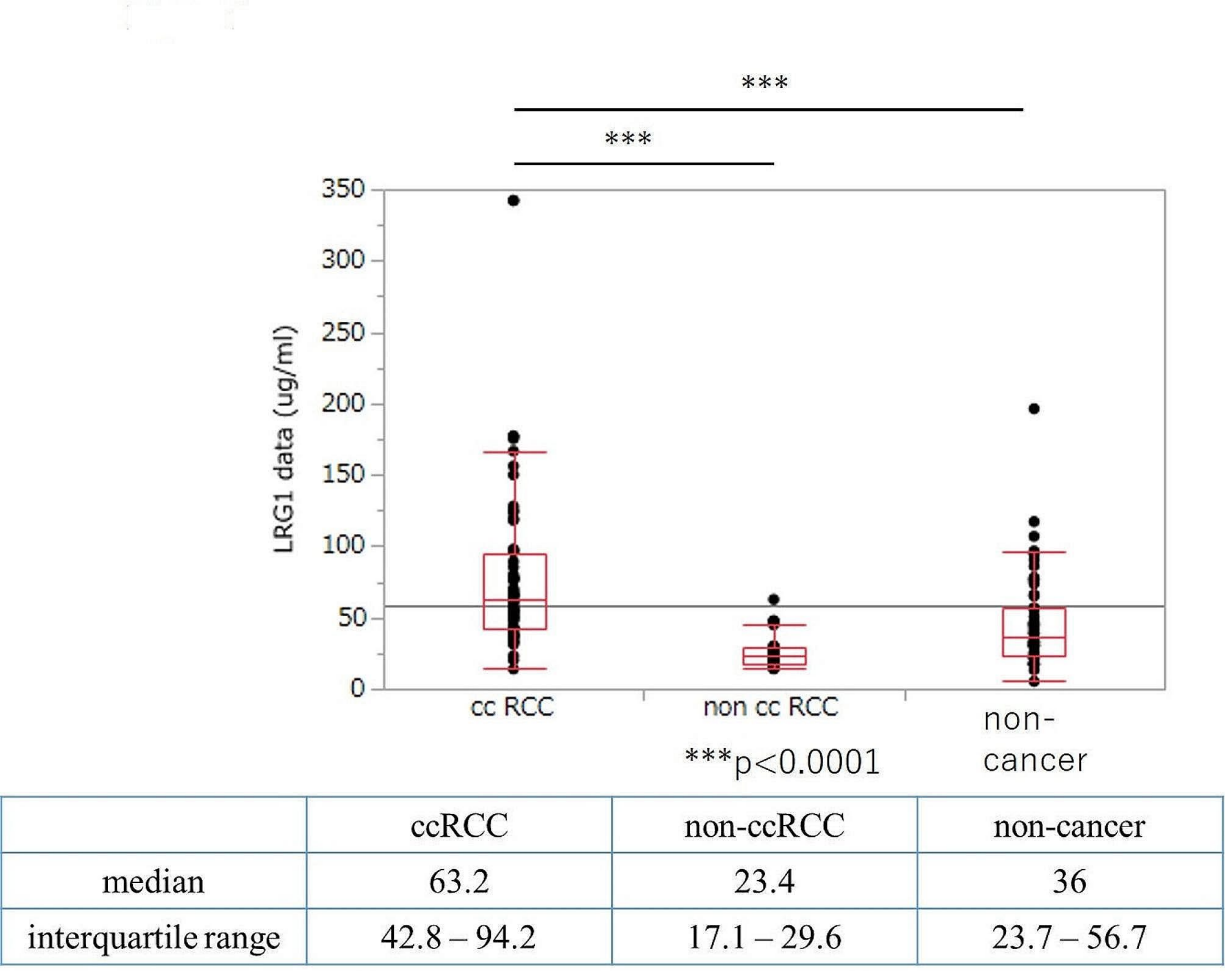
Measurements of serum LRG1 by enzyme-linked immunosorbent assay. Serum LRG1 levels in patients with ccRCC (*n* = 64), non-ccRCC (*n* = 22), and those without cancer (*n* = 63) were compared

Serum levels of LRG1 in patients with ccRCC (*n* = 64), non-ccRCC (*n* = 22), and without cancer (*n* = 63) were measured using ELISA. The median serum levels of LRG1, including the interquartile range, were 63.2 (42.8–94.2) µg/mL in ccRCC, 23.4 (17.7–29.6) µg/mL in non-ccRCC, and 36.0 (23.7–56.7) µg/mL in non-cancer patients, respectively (Fig. 2). The serum LRG1 level was significantly higher in patients with ccRCC than in those non-ccRCC (*P* < 0.001) or without cancer (*P* < 0.001). We confirmed that semi-quantification by western blotting and ELISA were significantly correlated (correlation coefficient, 0.85; 95% CI, 0.50–0.96) (data not shown).

### Relationship between serum LRG1 level and clinicopathological parameters in ccRCC

The predictor variables were analyzed for serum LRG1 levels in 61 of 64 ccRCC cases (the necessary data were lacking in three cases, which were omitted). Serum LRG1 levels were significantly associated with hemoglobin (*P* = 0.006), albumin (*P* = 0.008), corrected calcium (*P* = 0.014), C-reactive protein (CRP) (*P* = 0.009), neutrophil-to-lymphocyte ratio (NLR) (*P* = 0.005), clinical T stage (*P* = 0.015), clinical M stage (*P* = 0.046), and histological grade (*P* = 0.002). Multivariable analysis revealed that CRP level (*P* = 0.002), grade (*P* = 0.032), hypercalcemia (*P* = 0.023) and hemoglobin level (*P* = 0.037) were independent predictors of LRG1 levels (Table 2). In this study, multivariable analysis showed no significant correlation between CRP and serum LRG1 in patients with non-ccRCC. The results are shown in Supplementary Table 2.

**Table 2:** The relationship between serum LRG1 level and clinicopahtological paramerters in ccRCC

Variables	Univariable	Multivariable	95% CI
	p value	p value	
Hb	0.006	0.037	(-13.10 - -0.43)
LDH	0.647		
Alb	0.008	0.620	(-23.18 - 38.53)
Ca	0.014	0.023	(3.96 - 51.94)
CRP	0.009	0.002	(4.62 - 18.34)
Platlet	0.481		
NLR	0.005	0.200	(-2.26 - 10.56)
M / F	0.202		
clinical T1 or 2, 3	0.015	0.959	(-34.27 - 32.57)
clinical M1 or M0	0.046	0.247	(-75.63 - 19.93)
Grade 1,2 or 3	0.002	0.032	(4.83 - 99.65)

### Diagnostic performance of LRG1

**Fig. 3:**
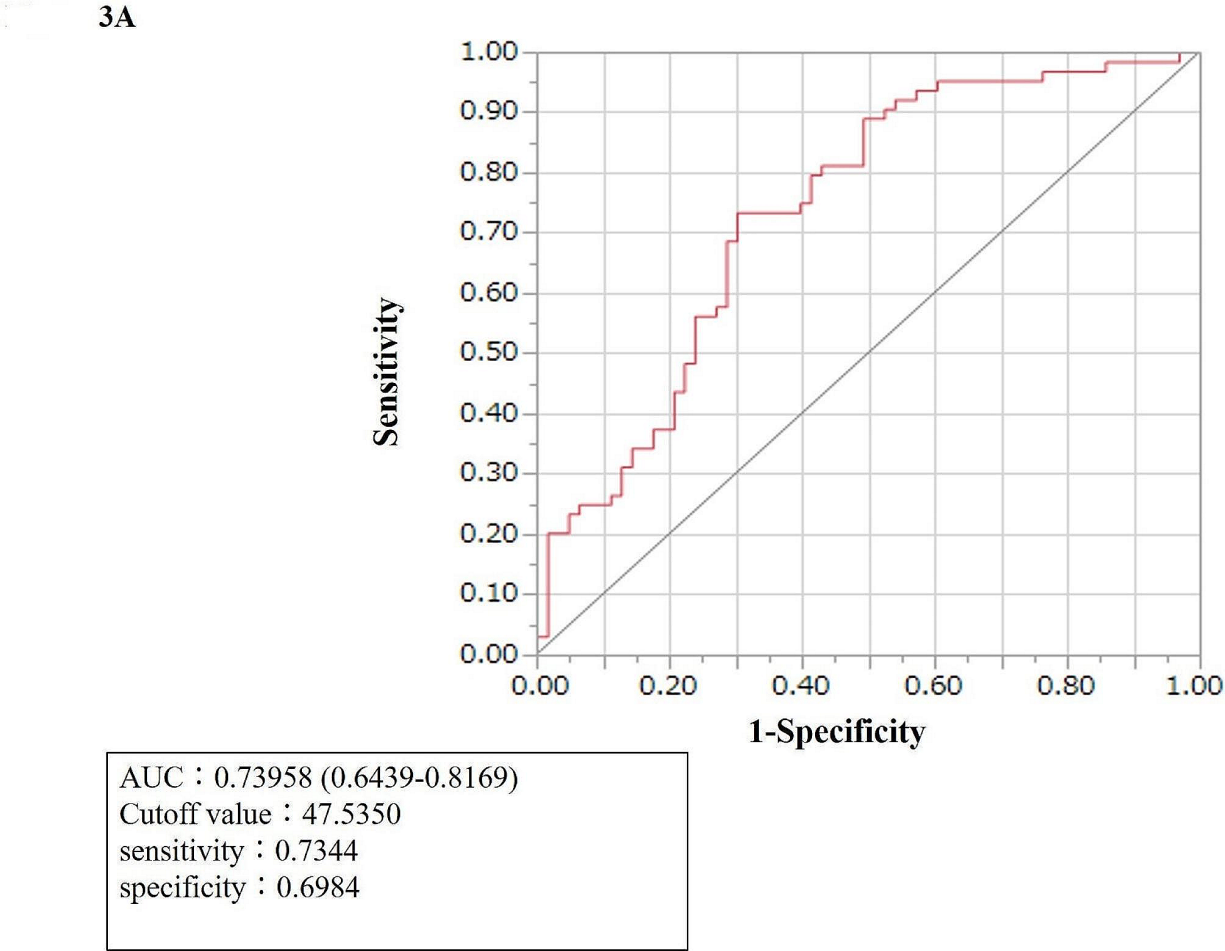
(A) ROC analysis of serum LRG1 to distinguish patients with ccRCC from those without cancer. (B) ROC analysis of serum LRG1 to distinguish patients with ccRCC from those with non-ccRCC

The area under the receiver operating characteristic curve (AUC) of serum LRG1 that was used for differentiating ccRCC from non-cancerous tissue was 0.74 (0.64–0.82), with the highest sensitivity (0.73) and specificity (0.70) observed at a cut-off value of 47.5 µg/mL (Fig. 3A). Meanwhile, the AUC of serum LRG1 that was used for distinguishing ccRCC from non-ccRCC was 0.91 (0.82–0.96), with the highest sensitivity (0.95) and specificity (0.82) obtained at a cut-off value of 31.2 µg/mL (Fig. 3B). We have performed multivariable analysis and confirmed that LRG1 is an independent factor for ccRCC differentiation. Shown as Supplementary Table 3. CRP was not a useful marker for differentiating between ccRCC and non-ccRCC.

## Discussion

In this study, it was revealed that serum LRG1 levels were significantly higher in patients with ccRCC compared to non-ccRCC and non-cancerous patients. As previous research suggests that LRG1 can induce angiogenesis [11], the increased level of serum LRG1 in ccRCC may partially explain the difference in vascularity between ccRCC and non-ccRCC, although VEGF and PDGF are the major factors associated with angiogenesis in RCC [18]. Furukawa et al. reported that increased serum LRG1 levels were related to the prognosis and clinical stage among patients with pancreatic cancer [19]. For esophageal squamous cell carcinoma, a significant relationship between LRG1 expression and T stage, M stage, and poor prognosis has been reported [20], suggesting that LRG1 is associated with the malignant potential of various cancers.

In recent years, several studies have highlighted the close association between inflammation and malignant potential [21]. Interleukin-6 and nuclear factor-kB have been shown to be representative molecules involved in the inflammatory response in RCC [22, 23]. CRP is a well-known marker of the acute phase of the systemic inflammatory response. Previous studies have revealed that CRP level is one of the most significant factors for predicting poor prognosis in both mRCC, and localized RCC [24–30]. In the current study, CRP levels, anemia, hypercalcemia, and grade were independent predictors of increased LRG1 levels. IL-6 expression is elevated in RCC and induces CRP production in hepatocytes [31]. IL-6 has also been reported to be significantly associated with anemia in RCC and induce hypercalcemia [32, 33]. LRG1 is also upregulated in HepG2 cells by IL-6 [34]. Therefore, LRG1 appears to be closely associated with IL-6-induced inflammation in RCC.

Regarding the roles of intracellular LRG1, several studies revealed that LRG1 regulates the TGF-β signalling pathway [11, 35, 36], serving a crucial role in tumor development. Cummings et al. reported that LRG1 can bind to and inhibit cytochrome c, an essential activator of cell apoptosis [37]. These findings suggest that intracellular LRG1 contributes to tumor growth, as previously described for ovarian cancer [13] and biliary tract cancer [38]. 

Since LRG1 was not detected in the culture media of ccRCC cell lines, LRG1 is considered to be released from non-RCC cells influenced by ccRCC, potentially contributing to angiogenesis in the ccRCC microenvironment. Similar to CRP production in the liver, IL-6 may induce the release of LRG1 from other cells [30]. Further studies are necessitated to identify the origin of LRG1 in the sera of patients with ccRCC.

Regarding diagnostic performance, the AUC of serum LRG1 for differentiating patients with ccRCC from non-cancerous patients was 0.73, and the diagnostic accuracy was classified as good [39]. The AUC of LRG1 for differentiating ccRCC from non-ccRCC was 0.91, and its diagnostic accuracy was classified as excellent [39]. Hence, LRG1 may be clinically useful for distinguishing ccRCC from non-ccRCC along with imaging modalities, especially when mRCC is present.

This study had a few limitations. First, the number of non-ccRCC patients was relatively small. Nevertheless, we demonstrated the excellent diagnostic accuracy of distinguishing ccRCC from non-ccRCC using the AUC [38]. Second, the effect of serum LRG1 levels on ccRCC prognosis could not be assessed due to insufficient patient numbers at each clinical stage for overall survival statistical analysis.

## Conclusions

In conclusion, Serum LRG1 levels were higher among patients with ccRCC than in non-ccRCC or non-cancerous patients. CRP, anemia, and hypercalcemia, all of which are related to inflammation and, tumor grade, are independent predictors of serum LRG1 levels. Therefore, LRG1 may serve as a serum marker reflecting the inflammatory and malignant potential of ccRCC. Furthermore, the AUC of LRG1 for distinguishing ccRCC from non-ccRCC was 0.91, indicating excellent diagnostic accuracy. Hence, LRG1 may be clinically useful in the differential diagnosis of ccRCC and non-ccRCC.

### Electronic supplementary material

Below is the link to the electronic supplementary material.

Supplementary Material 1

Supplementary Material 2

Supplementary Material 3

Supplementary Material 4

Supplementary Material 5

Supplementary Material 6

## Data Availability

Data supporting the findings of this study are available from the corresponding author upon request.

## Abbreviations

LRG1: leucine-rich-alpha-2-glycoprotein 1
RCC: renal cell carcinoma
ccRCC: clear cell renal cell carcinoma
mRCC: metastatic renal cell carcinoma
TKI: tyrosine kinase inhibitor
ELISA: enzyme-linked immunosorbent assay
IO: immune-oncology
CRP: C-reactive protein
NLR: neutrophil-to-lymphocyte ratio
AUC: area under the receiver operating characteristic curve

## References

[1] Rini BI, Campbell SC, Escudier B (2009). Renal cell carcinoma. Lancet (London England).

[2] Ljungberg B (2007). Prognostic markers in renal cell carcinoma. Curr Opin Urol.

[3] Motzer RJ, Hutson TE, Tomczak P, Michaelson MD, Bukowski RM, Rixe O, Oudard S, Negrier S, Szczylik C, Kim ST, Chen I, Bycott PW, Baum CM, Figlin RA (2007). Sunitinib versus interferon alfa in metastatic renal-cell carcinoma. N Engl J Med.

[4] Motzer RJ, Escudier B, McDermott DF, George S, Hammers HJ, Srinivas S, Tykodi SS, Sosman JA, Procopio G, Plimack ER, Castellano D, Choueiri TK, Gurney H, Donskov F, Bono P, Wagstaff J, Gauler TC, Ueda T, Tomita Y, Schutz FA, Kollmannsberger C, Larkin J, Ravaud A, Simon JS, Xu LA, Waxman IM, Sharma P (2015). Nivolumab versus Everolimus in Advanced Renal-Cell Carcinoma. N Engl J Med.

[5] de Velasco G, McKay RR, Lin X, Moreira RB, Simantov R, Choueiri TK (2017). Comprehensive Analysis of Survival Outcomes in Non-clear Cell Renal Cell Carcinoma patients treated in clinical trials. Clin Genitourin Cancer.

[6] Haupt H, Baudner S (1977). [Isolation and characterization of an unknown, leucine-rich 3.1-S-alpha2-glycoprotein from human serum (author’s transl)]. Hoppe-Seyler’s Z fur Physiol Chemie.

[7] Kobe B, Kajava AV (2001). The leucine-rich repeat as a protein recognition motif. Curr Opin Struct Biol.

[8] Saito K, Tanaka T, Kanda H, Ebisuno Y, Izawa D, Kawamoto S, Okubo K, Miyasaka M (2002). Gene expression profiling of mucosal addressin cell adhesion molecule-1 + high endothelial venule cells (HEV) and identification of a leucine-rich HEV glycoprotein as a HEV marker. J Immunol (Baltimore Md: 1950).

[9] Weivoda S, Andersen JD, Skogen A, Schlievert PM, Fontana D, Schacker T, Tuite P, Dubinsky JM, Jemmerson R (2008). ELISA for human serum leucine-rich alpha-2-glycoprotein-1 employing cytochrome c as the capturing ligand. J Immunol Methods.

[10] Thompson FC (2007). The Vietnam War added a motive to go on studying. Nature.

[11] Wang X, Abraham S, McKenzie JAG, Jeffs N, Swire M, Tripathi VB, Luhmann UFO, Lange CAK, Zhai Z, Arthur HM, Bainbridge J, Moss SE, Greenwood J (2013). LRG1 promotes angiogenesis by modulating endothelial TGF-beta signalling. Nature.

[12] Andersen JD, Boylan KL, Jemmerson R, Geller MA, Misemer B, Harrington KM, Weivoda S, Witthuhn BA, Argenta P, Vogel RI, Skubitz AP (2010). Leucine-rich alpha-2-glycoprotein-1 is upregulated in sera and tumors of ovarian cancer patients. J Ovarian Res.

[13] Liu Y, Luo X, Hu H, Wang R, Sun Y, Zeng R, Chen H (2012). Integrative proteomics and tissue microarray profiling indicate the association between overexpressed serum proteins and non-small cell lung cancer. PLoS ONE.

[14] Uen YH, Lin KY, Sun DP, Liao CC, Hsieh MS, Huang YK, Chen YW, Huang PH, Chen WJ, Tai CC, Lee KW, Chen YC, Lin CY (2013). Comparative proteomics, network analysis and post-translational modification identification reveal differential profiles of plasma con A-bound glycoprotein biomarkers in gastric cancer. J Proteom.

[15] Kakisaka T, Kondo T, Okano T, Fujii K, Honda K, Endo M, Tsuchida A, Aoki T, Itoi T, Moriyasu F, Yamada T, Kato H, Nishimura T, Todo S, Hirohashi S (2007). Plasma proteomics of pancreatic cancer patients by multi-dimensional liquid chromatography and two-dimensional difference gel electrophoresis (2D-DIGE): up-regulation of leucine-rich alpha-2-glycoprotein in pancreatic cancer. J Chromatogr B Anal Technol Biomedical life Sci.

[16] Wu RS, Yu CS, Liu KC, Huang HY, Ip SW, Lin JP, Chueh FS, Yang JS, Chung JG (2012). Citosol (thiamylal sodium) triggers apoptosis and affects gene expressions of murine leukemia RAW 264.7 cells. Hum Exp Toxicol.

[17] Leslie H, Sobin, Mary K, Gospodarowicz. Christian Wittekind. TNM Classification of Malignant Tumours, 7th Edition. 2011.

[18] Zerbini G, Lorenzi M, Palini A (2008). Tumor angiogenesis. N Engl J Med.

[19] Furukawa K, Kawamoto K, Eguchi H, Tanemura M, Tanida T, Tomimaru Y, Akita H, Hama N, Wada H, Kobayashi S, Nonaka Y, Takamatsu S, Shinzaki S, Kumada T, Satomura S, Ito T, Serada S, Naka T, Mori M, Doki Y, Miyoshi E, Nagano H (2015). Clinicopathological significance of leucine-rich alpha2-Glycoprotein-1 in Sera of patients with pancreatic Cancer. Pancreas.

[20] Wang Y, Xing Q, Chen X, Wang J, Guan S, Chen X, Sun P, Wang M, Cheng Y. The clinical prognostic value of LRG1 in esophageal squamous cell carcinoma. Curr Cancer Drug Targets. 2019. 10.2174/1568009619666190204095942 30714525

[21] Coussens LM, Werb Z (2002). Inflammation and cancer. Nature.

[22] Blay JY, Rossi JF, Wijdenes J, Menetrier-Caux C, Schemann S, Negrier S, Philip T, Favrot M (1997). Role of interleukin-6 in the paraneoplastic inflammatory syndrome associated with renal-cell carcinoma. Int J Cancer.

[23] Oya M, Takayanagi A, Horiguchi A, Mizuno R, Ohtsubo M, Marumo K, Shimizu N, Murai M (2003). Increased nuclear factor-kappa B activation is related to the tumor development of renal cell carcinoma. Carcinogenesis.

[24] Heikkila K, Ebrahim S, Lawlor DA (2007). A systematic review of the association between circulating concentrations of C reactive protein and cancer. J Epidemiol Commun Health.

[25] Wu Y, Fu X, Zhu X, He X, Zou C, Han Y, Xu M, Huang C, Lu X, Zhao Y (2011). Prognostic role of systemic inflammatory response in renal cell carcinoma: a systematic review and meta-analysis. J Cancer Res Clin Oncol.

[26] Johnson TV, Abbasi A, Owen-Smith A, Young A, Ogan K, Pattaras J, Nieh P, Marshall FF, Master VA (2010). Absolute preoperative C-reactive protein predicts metastasis and mortality in the first year following potentially curative nephrectomy for clear cell renal cell carcinoma. J Urol.

[27] Komai Y, Saito K, Sakai K, Morimoto S (2007). Increased preoperative serum C-reactive protein level predicts a poor prognosis in patients with localized renal cell carcinoma. BJU Int.

[28] Omae K, Kondo T, Tanabe K (2015). High preoperative C-reactive protein values predict poor survival in patients on chronic hemodialysis undergoing nephrectomy for renal cancer. Urol Oncol.

[29] Hsiao W, Herrel LA, Yu C, Kattan MW, Canter DJ, Carthon BC, Ogan K, Master VA (2015). Nomograms incorporating serum C-reactive protein effectively predict mortality before and after surgical treatment of renal cell carcinoma. Int J Urology: Official J Japanese Urol Association.

[30] Naito S, Kinoshita H, Kondo T, Shinohara N, Kasahara T, Saito K, Takayama T, Masumori N, Takahashi W, Takahashi M, Terachi T, Ozono S, Naito S, Tomita Y (2013). Prognostic factors of patients with metastatic renal cell carcinoma with removed metastases: a multicenter study of 556 patients. Urology.

[31] Kedar I, Mermershtain W, Ivgi H (2004). Thalidomide reduces serum C-reactive protein and interleukin-6 and induces response to IL-2 in a fraction of metastatic renal cell cancer patients who failed IL-2-based therapy. Int J Cancer.

[32] Falkensammer CE, Thurnher M, Leonhartsberger N, Ramoner R (2011). C-reactive protein is a strong predictor for anaemia in renal cell carcinoma: role of IL-6 in overall survival. BJU Int.

[33] Weissglas MG, Schamhart DH, Lowik CW, Papapoulos SE, Theuns HM, Kurth KH (1997). The role of interleukin-6 in the induction of hypercalcemia in renal cell carcinoma transplanted into nude mice. Endocrinology.

[34] Shirai R, Hirano F, Ohkura N, Ikeda K, Inoue S (2009). Up-regulation of the expression of leucine-rich alpha(2)-glycoprotein in hepatocytes by the mediators of acute-phase response. Biochem Biophys Res Commun.

[35] Song W, Wang X (2015). The role of TGFbeta1 and LRG1 in cardiac remodelling and heart failure. Biophys Rev.

[36] Lynch J, Fay J, Meehan M, Bryan K, Watters KM, Murphy DM, Stallings RL (2012). MiRNA-335 suppresses neuroblastoma cell invasiveness by direct targeting of multiple genes from the non-canonical TGF-beta signalling pathway. Carcinogenesis.

[37] Cummings C, Walder J, Treeful A, Jemmerson R (2006). Serum leucine-rich alpha-2-glycoprotein-1 binds cytochrome c and inhibits antibody detection of this apoptotic marker in enzyme-linked immunosorbent assay. Apoptosis: Int J Program cell Death.

[38] Sandanayake NS, Sinclair J, Andreola F, Chapman MH, Xue A, Webster GJ, Clarkson A, Gill A, Norton ID, Smith RC, Timms JF, Pereira SP (2011). A combination of serum leucine-rich alpha-2-glycoprotein 1, CA19-9 and interleukin-6 differentiate biliary tract cancer from benign biliary strictures. Br J Cancer.

[39] Simundic AM (2009). Measures of diagnostic accuracy: Basic definitions. Ejifcc.

