# Supplementary figures and images for: Increased level of serum leucine-rich-alpha-2-glycoprotein 1 in patients with clear cell renal cell carcinoma
Source: BMC Urol. 2024 Apr 24;24:94. doi: 10.1186/s12894-024-01481-0 (PMC11040933; doi:10.1186/s12894-024-01481-0)

## Slide 1
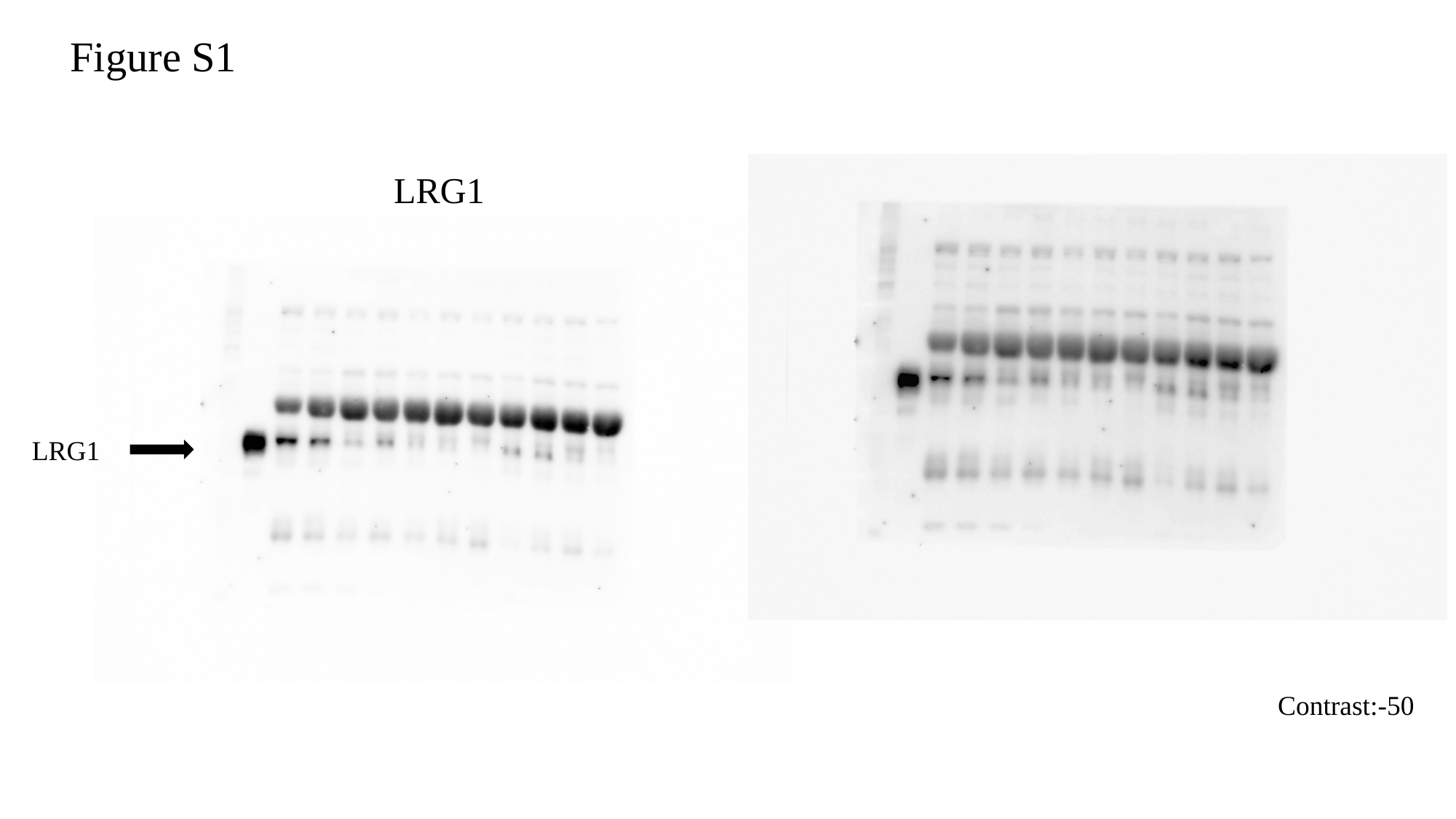

Figure S1
Contrast:-50
LRG1
LRG1

Supplement: Supplementary file 2 — Supplementary Material 2 [file 12894_2024_1481_MOESM2_ESM.pptx]

## Slide 1
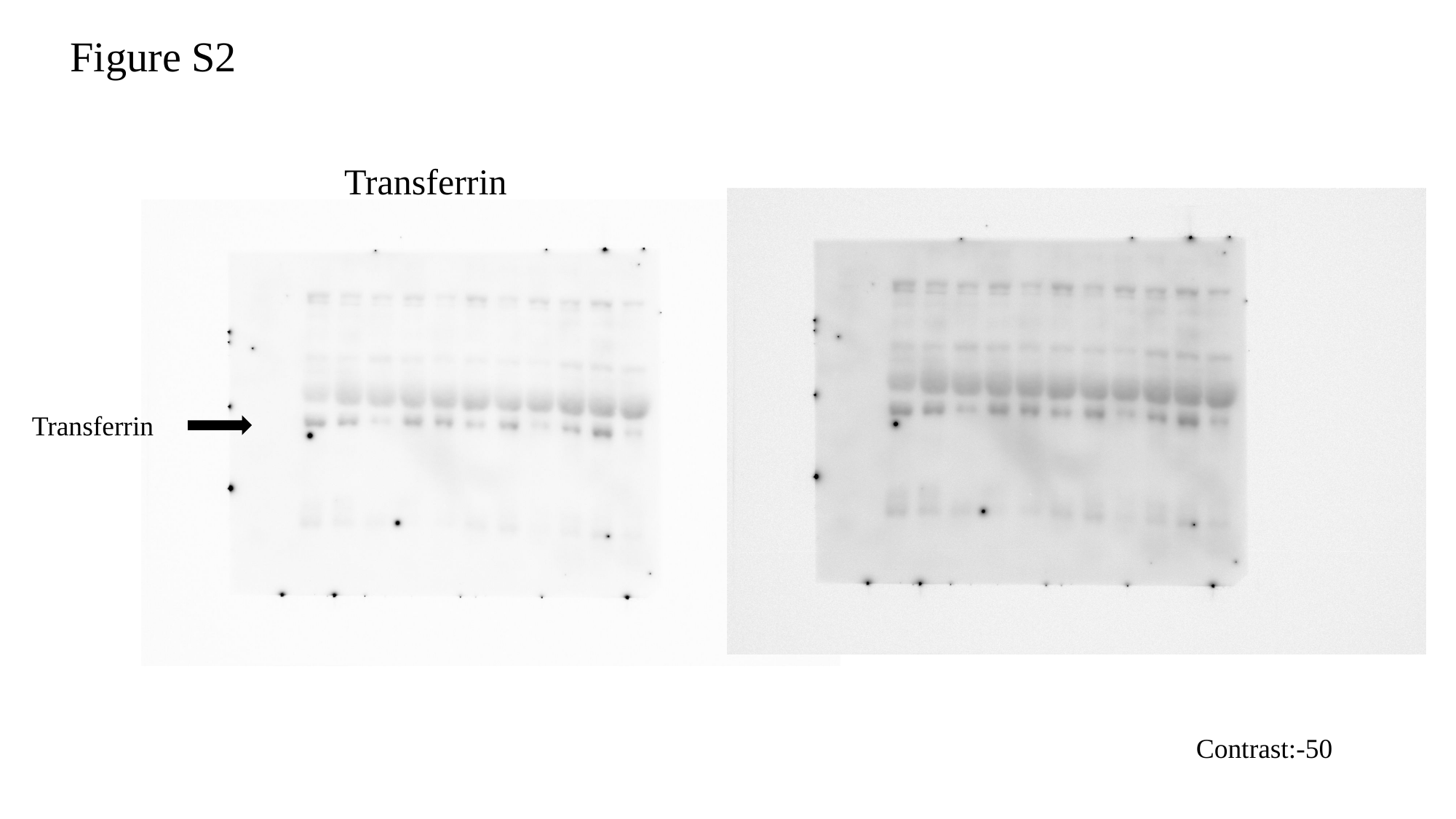

Figure S2
Transferrin
Transferrin
Contrast:-50

Supplement: Supplementary file 3 — Supplementary Material 3 [file 12894_2024_1481_MOESM3_ESM.pptx]
