# Supplementary material for: Increased level of serum leucine-rich-alpha-2-glycoprotein 1 in patients with clear cell renal cell carcinoma
Source: BMC Urol. 2024 Apr 24;24:94. doi: 10.1186/s12894-024-01481-0 (PMC11040933; doi:10.1186/s12894-024-01481-0)
Supplement: Supplementary file 4 — Supplementary Material 4 [file 12894_2024_1481_MOESM4_ESM.docx]

**Supplementary Information**

**Figure legends**

**Figure S1.** Western blotting was utilized to determine if LRG1 was present in RCC serum.

**Figure S2.** Transferrin was utilized as an internal control.
